# How SLX4 cuts through the mystery of HIV-1 Vpr-mediated cell cycle arrest

**DOI:** 10.1186/s12977-014-0117-5

**Published:** 2014-12-11

**Authors:** Marie-Lise Blondot, Loic Dragin, Hichem Lahouassa, Florence Margottin-Goguet

**Affiliations:** Inserm, U1016, Institut Cochin, Paris, France; Cnrs, UMR8104, Paris, France; Université Paris Descartes, Sorbonne Paris Cité, France

**Keywords:** HIV, Vpr, SLX4, Endonucleases, Innate immune response, Cell cycle, DNA repair

## Abstract

Vpr is one of the most enigmatic viral auxiliary proteins of HIV. During the past twenty years, several activities have been ascribed to this viral protein, but one, its ability to mediate cell cycle arrest at the G2 to M transition has been the most extensively studied. Nonetheless, the genuine role of Vpr and its pathophysiological relevance in the viral life cycle have remained mysterious. Recent work by Laguette *et al*. (Cell 156:134-145, 2014) provides important insight into the molecular mechanism of Vpr-mediated G2 arrest. This study highlights for the first time how Vpr recruits the SLX4 endonuclease complex and how Vpr-induced inappropriate activation of this complex leads to G2 arrest. Here, we will discuss these findings in the light of previous work to show how they change the view of Vpr’s mechanism of action. We will also discuss how these findings open new questions towards the understanding of the biological function of Vpr regarding innate immune sensing.

## Introduction

HIV type 1 and type 2 primate lentiviruses encode a set of auxiliary proteins that differ between the two lentiviral lineages. Vpr, Nef and Vif are expressed by both the HIV-1/SIVcpz and HIV-2/SIVsmm lineages, while Vpu is specific to the former and Vpx to the latter. These auxiliary proteins play a crucial role at the host-virus interface by modulating the intracellular micro-environment in favor of viral infection and dissemination [1]. So far, much work conducted to decipher their mechanism of action has helped to understand the molecular steps faced by HIV during the viral life cycle. Most HIV auxiliary proteins use the same strategy, *i.e*. hijacking a ubiquitin ligase complex, to target cellular proteins, the so-called restriction factors, for proteasome-mediated degradation. This is how HIV-1 Vpu, Vif or HIV-2/SIVsmm Vpx counteract tetherin/BST-2, APOBEC3G and SAMHD1 respectively [2]. Each cellular target displays specific and potent antiviral activity; *de facto*, some *in vitro* systems, in which the restriction factor is expressed, can recapitulate the dependence of infection on the viral protein. Alike Vpu, Vif or Vpx, we and others have previously shown that HIV-1 Vpr is also able to recruit a ubiquitin ligase complex, but the significance of such an interaction has remained unknown [3].

Expression of Vpr in cycling cells triggers cell cycle arrest at the G2 phase, prior to mitosis. Vpr-mediated G2 arrest was revealed in 1995 by several groups [4-8] and represents the most widely described property of the viral protein. However, the biological significance of this activity towards infection has been unclear for years given that efficient viral replication does not require Vpr in dividing cells. Paradoxically, a lack of Vpr slightly affects viral infection in macrophages that do not divide [9-12], suggesting that Vpr-mediated cell cycle arrest is irrelevant to its role towards infection in these cells.

The recent study by Laguette and collaborators [13] provides new information on the G2-arrest mechanism induced by Vpr by highlighting its connection with the SLX4 endonuclease complex (SLX4com), and in addition, reports a potential anti-immune role for Vpr *via* this interaction.

## Review

### Hijacking DCAF1/VprBP in S-phase to induce cell cycle arrest at the G2/M transition

In an attempt to decipher the molecular mechanisms underlying Vpr-mediated G2 arrest, several studies have reported that this activity depends on the DNA damage checkpoint network. This pathway involves the activation of ATR (Ataxia Telangiectasia-mutated and Rad3-related) and Chk1 kinases and ends-up with the inactivation of the cyclinB-Cdk1 complex, which governs mitosis entry [14-19]. In cooperation with its downstream effectors, ATR preserves genome stability, in particular by helping stalled replication forks to proceed following genotoxic stress [20]. Vpr-mediated induction of replication stress was observed in transformed cell lines but also in primary CD4+ T cells and correlates with the accumulation of replication protein A, which binds single stranded DNA following replication stress [19]. In good agreement, the pathway used by Vpr was also shown to result from an S phase-dependent mechanism and to involve association of Vpr with chromatin [19,21-23]. Yet, the precise mechanism by which Vpr caused DNA replication stress and the role of this stress during the course of the infection has remained elusive.

In 2007, Vpr binding protein (VprBP), later on renamed DCAF1 for DDB1-Cul4A-associated factor 1 (DDB1: DNA damage-binding protein 1, Cul4A: Cullin 4A) was recognized as a critical host factor in the ability of Vpr to trigger cell cycle arrest [22,24-29]. Prior to this, DCAF1/VprBP was first identified as a cellular protein binding to Vpr with high affinity [30] and ten years after, characterized as an adaptor of DDB1-Cul4 ubiquitin ligases [31-34]. The data collectively obtained by the different groups supported a model [22,24-28] in which Vpr simultaneously recruits the Cul4A ubiquitin ligase through DCAF1 and a so far unknown cellular protein required for entry into mitosis. As a result, the Vpr target protein was supposed to be ubiquitinated and degraded to subsequently trigger G2 arrest. Vpr itself was predicted to escape from DCAF1-induced degradation and even to be stabilized by its association with the Cul4A-DDB1 ubiquitin ligase [35]. In view of the model of ubiquitin ligase hijacking for the benefit of the viral cycle, we and others have hypothesized that the function of Vpr was dependent on the degradation of a specific host protein rather than the resulting G2 arrest that could be a side effect in cycling cells. In this case, we expected the host target of Vpr to be a negative factor for viral replication [3]. For many investigators, identification of the host protein(s) targeted for degradation by Vpr became a priority toward understanding the genuine function of this viral protein.

### Revisiting Vpr-mediated G2 arrest mechanism within the context of SLX4

**Figure 1:**
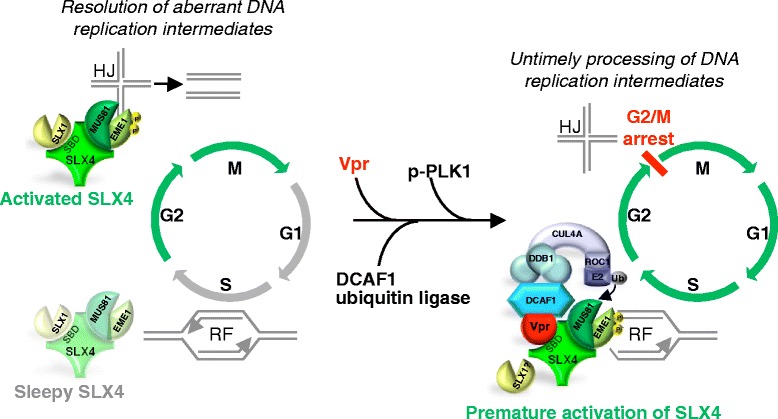
**Model for Vpr-mediated G2 arrest through remodeling of the SLX4 complex.** In a physiological contex activation of the SLX4 complex (SLX4com) intervenes to allow the resolution of DNA replication intermediates, such as Holliday Junctions (HJ), found in collapsed replication forks after DNA replication (phases of SLX4 activation are symbolized by green arrows). This leads to a proper G2/M phase transition (left panel). HIV-1 Vpr protein interacts with SLX4 on its SLX1 binding domain, the CUL4A–DDB1^DCAF1^ ubiquitin ligase and PLK1/p-PLK1. These interactions lead to MUS81 ubiquitination, and phosphorylation of EME1 by p-PLK1, resulting in aberrant activation of SLX4com. This inappropriate activation perturbs progression of ongoing replication forks (RF) in S phase and the resolution of abnormal DNA replication intermediates. As a consequence, cells arrest at the G2 phase of the cell cycle (right panel).

Following tandem affinity purification, immunoprecipitation and mass spectrometry, Laguette *et al*. identified structure-specific endonucleases (SSEs), namely ERCC1-ERCC4/XPF and MUS81-EME1, together with the SLX4 scaffold protein as new Vpr interacting partners [13]. Glycerol gradient sedimentation and co-immunoprecipitation studies confirmed that SLX4 subunits, DCAF1 and Vpr assemble into a single complex. SLX4, also known as BTBD12, was renamed FANCP, when biallelic mutations in the *SLX4* gene were associated with Fanconi anemia (FA), an autosomal recessive genetic disorder characterized by congenital abnormalities, bone marrow failure and cancer susceptibility [36-39]. SLX4 provides a molecular platform to form a complex (SLX4com) with several SSEs, ERCC1-ERCC4/XPF, MUS81-EME1, but also SLX1, and stimulates their activities to coordinate the repair of specific replication-born double strand breaks (DSBs) and collapsed replication forks [38-40]. MUS81-EME1 and SLX1 are specifically dedicated to the resolution of replication-induced X-shaped DNA structures, the so-called Holliday Junctions, during homologous recombination [41-43]. These SSEs are submitted to strict regulations along the cell cycle to ensure the formation of an active complex at the right time and subsequently the removing of inadequate DNA recombination intermediates before chromosome segregation [41,44-48] (Figure 1). Laguette *et al*. observed that Vpr induces a slight decrease in EME1 and MUS81 expression and an increase in the levels of polo-like kinase 1 (PLK1) and phospho-PLK1, a kinase that activates the endonuclease activity of EME1 through its phosphorylation [39,45]. In parallel, Vpr caused a remodeling of SLX4com through its association with phospho-EME1 and the DCAF1 ubiquitin ligase (Figure 1). Importantly, time course experiments led to the conclusion that Vpr-induced activation of SLX4com precedes G2/M arrest and is not a consequence of the cell cycle arrest *per se*. In addition, SLX4 purified in the presence of Vpr had increased cleavage activity toward radiolabelled DNA substrates as compared to the endonuclease in the absence of Vpr. Interestingly, Vpr-mediated increase of SLX4com activity was abolished when MUS81 expression was silenced. Importantly also, downregulation of SLX4, EME1 or MUS81 expression inhibited Vpr-mediated G2 arrest, which has been recently confirmed by Berger et al. [49]. Collectively, the data support a model in which Vpr induces the premature activation of SLX4com, leading to replication stress as asserted by the appearance of FANCD2 foci [50]. Consequently, the abnormal cleavage of DNA replication intermediates would presumably cause a G2 arrest. It is unclear yet whether this process is related to previous observations suggesting that Vpr induces double-strand breaks (DSB) formation or that MUS81 plays a role in the formation of such breaks in response to the inhibition of replication [51,52]. Moreover, the interaction of SLX4com with chromatin in the presence or not of Vpr remains to be evaluated.

The Vpr-binding protein DCAF1 was also present in association with SLX4 and this interaction was enhanced with Vpr. Given its role in Vpr-mediated G2 arrest, the role of DCAF1 in SLX4com activation was further investigated. Silencing of DCAF1 reduced the accumulation of PLK1 and Phospho-PLK1 seen in the presence of Vpr and inhibited the reduction in MUS81 levels. In addition, more ubiquitinated MUS81 species were detected with wt Vpr, by comparing with the DCAF1-binding deficient Vpr mutant (Q65R), which did not induce FANCD2 foci accumulation. Nonetheless, whether the proteasome pathway is involved in this process remains to be investigated with the use, for example, of proteasome inhibitors. Altogether the results suggest that Vpr uses DCAF1 for SLX4com activation and reduction of MUS81 levels.

Vpr-mediated downregulation of MUS81 expression seems relatively inefficient. One explanation could be that degradation occurs in a short window of cell cycle progression, just before G2/M, making it difficult to highlight in asynchronous cells. Moreover, how degradation of MUS81 intervenes in the process of SLX4com activation remains unclear. As a viral protein hijacking a ubiquitin ligase, Vpr was initially thought to trigger the degradation of an antiviral factor. Here, the ubiquitination and the downregulation of MUS81 by Vpr could lead to envision it as a potential antiviral factor counteracted by Vpr and whose degradation would lead to G2 arrest. However, this is not the case, MUS81 depletion does not mimic a G2 arrest phenotype and MUS81 or SLX4 depletion reduces viral infection, which indicates their positive role for the viral life cycle. Nonetheless, it is intriguing that MUS81 is both required for G2 arrest and also inactivated by Vpr through DCAF1. Further studies will help to understand the orchestration of these different events.

Though the SLX1 SSE does not co-purify with Vpr and SLX4, SLX1 seems to participate in Vpr-mediated G2 arrest. Indeed, depletion of SLX1 reduces the ability of Vpr to mediate G2 arrest. In addition, purified SLX4com isolated from Vpr expressing cells still processes SLX1 substrates. Of note, Vpr interacts with the SLX1 binding domain of SLX4, which may indicate that interaction of SLX4 with SLX1 and with Vpr are exclusive. These apparently contradictory findings may reflect that a series of complex and successive events are involved in Vpr-mediated SLX4 activation.

Recent work by Berger et al. further supports a role of SLX4, MUS81 and EME1 in Vpr-mediated G2 arrest [49]. Interestingly, SIV Vpr alleles competent for G2 arrest in human cells interact with human SLX4, but not alleles incompetent for G2 arrest in those cells. SIV Vpr alleles competent for G2 arrest in Grivet cells, but not in human cells, interact with simian SLX4. This species-specificity in Vpr-mediated G2 arrest supports the idea of a central role of SLX4 in Vpr function.

Altogether the results by Laguette *et al.* support a model in which Vpr forces activation of the SLX4 complex, rather than supporting the previous model in which Vpr eliminates an antiviral factor. Nonetheless, we are tempted to put forward the hypothesis that the two models could be reconciled in a model in which Vpr would for example induce the degradation of an inhibitor of SLX4 activation that has potent antiviral activity.

### Vpr and escape from immune sensing

**Figure 2:**
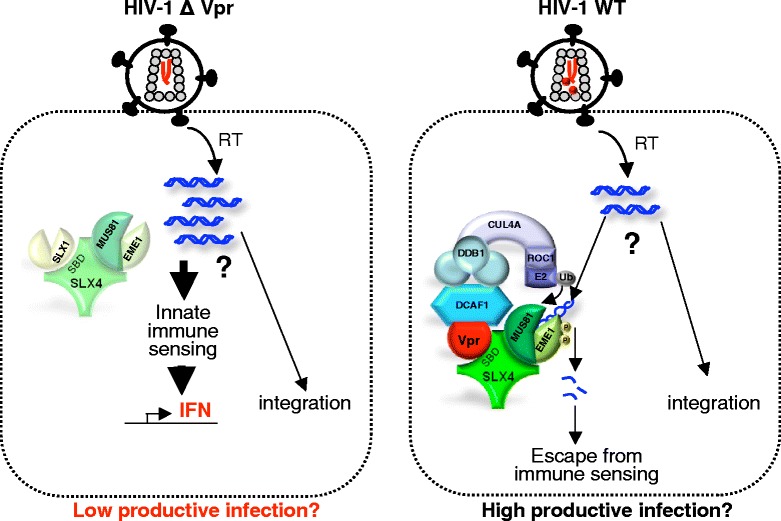
**Model for Vpr-mediated evasion from immune sensing.** The model envisioned by Laguette *et al.* proposes that HIV DNA forms accumulate following infection by a Vpr-deleted HIV-1 virus (HIV-1∆Vpr). These forms, that are not characterized, are sensed by a DNA immune sensor to generate an IFN response (left panel). A Vpr encoding HIV-1 virus would instead force the SLX4com endonuclease activity. The subsequent processing of extra reverse transcription products would help to prevent their accumulation and allow escape from immune sensing (right panel). How does SLX4com distinguish between the unproductive and the productively infecting RT intermediates is unknown. In addition, whether Vpr increases the efficiency of viral infection by activating SLX4 has not been investigated yet.

Persistent viruses such as HIV are known to interfere with the overall immune host response [53]. The role of Vpr in this context has been studied by several groups. Namely, two reports have previously demonstrated that Vpr decreases the expression of IRF3, an essential protein for interferon (IFN) beta production in response to viral infection [54,55]. Furthermore, numerous immunosuppressive activities of Vpr have been identified, including its capacity to perturb the Th1 lymphocytes activity or the Treg homeostasis [56,57]. Altogether, these studies highlight a new capacity of Vpr to participate in the viral persistence by hindering the appropriate cooperation between immune cells. In Laguette *et al*., deletion of Vpr induces a 2 to 3 fold increase of the expression of mRNA of the type 1 interferon response (IFNα, IFNβ and MxA) upon HIV-1 infection in HeLa cells, supporting the notion that Vpr could contribute to HIV-1 escape from innate immune sensing. Nonetheless, whether reduction in IFN-related mRNAs in the presence of Vpr is related to SLX4 remains difficult to assess since deletion of SLX4 or MUS81 alone leads to a huge increase of the same mRNAs. Interestingly though, the authors showed that HIV-1 DNA products of reverse transcription are pulled down with epitope-tagged SLX4 specifically in the presence of Vpr and that reduction of SLX4 expression leads to an increase in HIV-1 DNA levels. These results suggest that Vpr could help to escape immune sensing by inducing the digestion of viral DNA through SLX4com activation (Figure 2).

Surprisingly, the increase in HIV-1 DNA levels under SLX4 knock-down is not associated with an increase in HIV-1 infection, however it should be stressed that the forms accumulated under SLX4 knock-down are not fully characterized yet and could correspond to abortive products of reverse transcription. It is also puzzling that no RT product is detected in the SLX4 immunoprecipitate a few hours after infection as we could expect from previous studies [58]. Given the presence of SLX4 into the nucleus, its association with 1/2LTR circles or even proviruses would be worth to look at deeply.

In a model in which SLX4 would play a role in the escape from immune sensing by digesting viral DNA, depletion of SLX4 should reduce viral infection. Accordingly, cells from FANCP patients or MEF cells depleted for SLX4 displayed high levels of IFNα, IFNβ and Mxa mRNA and a decreased permissivity to viral transduction. Of note, the decrease in HIV-1 infection under SLX4 depletion was seen in patient samples but not in HeLa cells depleted for SLX4 or MUS81. Why the high increase in IFN-related mRNAs in HeLa cells does not correlate with an inhibition of HIV-1 transduction in these same cells is puzzling. In other circumstances, production of IFN inhibits HIV-1 transduction in single-cycle infection experiments, for instance following depletion of the TREX1 endonuclase [59]. Several hypotheses can account for the lack of effect of Vpr on HIV-1 infection in HeLa cells. Though an effect on IFN-related mRNA levels is detected, it is likely that this effect does not lead to efficient interferon production, as it could be the case in other primary cells. Alternatively, the production of IFN might be too weak to have a negative effect on single-round infection, but would be cumulative in a spreading infection using replicative viruses. In this setting also, it is likely that HeLa cells do not have all the connections that allow efficient IFN signaling for subsequent HIV-1 infection inhibition. Surprisingly, a recent report has provided evidence that HIV-1 Vpr induces Interferon-Stimulated Genes (ISG) and helps to activate innate immune response in non-infected macrophages [60]. These apparent discrepancies may result from the use of different cellular systems, non-infected macrophages *versus* infected Hela cells. Further experiments by additional investigators will help to draw a clearer picture of what is going on under Vpr treatment in different cell types.

Previous work has demonstrated how a subset of Vpr proteins from lentiviruses lacking the auxiliary protein Vpx were still able to degrade the antiviral factor SAMHD1 as well as Vpx from HIV-2/SIVsmm/SIVmac [61-63]. Alike SLX4, SAMHD1 modulates the DNA damage response [64]. Mutations of SAMHD1 are found in the Aicardi-Goutières syndrome (AGS), a rare autosomal recessive genetic encephalopathy characterized by high levels of IFN leading to chronic inflammation [65]. Several studies indicate that SAMHD1 limits proinflammatory cytokine production and immune detection [65-70]. Inhibition of the immune sensing of HIV-1 through SAMHD1 results from the dNTPase activity of the protein, which contributes to the removal of nucleotides essential for viral cDNA synthesis in myeloid cells and quiescent CD4+ T cells, but could also potentially results from its recently identified RNAse activity [71-78]. Nonetheless, HIV-2/SIVsmm/SIVmac and a subset of other lentiviruses have kept the ability to degrade SAMHD1, in order to increase dNTP levels for efficient reverse transcription, even at the expense of the possibility to escape immune detection. Strikingly, HIV-1 Vpr and HIV-2/SIVsmm Vpx seem to function in opposite directions with respect to immune sensing. Altogether, by having no Vpx but one Vpr, HIV-1 has significant assets to escape immune detection compared to HIV-2. In addition, HIV-2 capsid harbors a major determinant for viral cDNA sensing, while HIV-1 capsid has been shown to prevent sensing or even to bind specific cofactors to evade immune detection [78-80]. Differences in escape from immune sensing likely contribute to the different pathogenic outcomes associated with the two viruses, HIV-1 being far more pathogenic than HIV-2 [81].

Other host proteins than SAMHD1 contribute to limit the sensing of viral nucleic acids, including RNASEH2, which degrades DNA:RNA hybrids or TREX1, which eliminates viral DNA in excess [82-85]. Importantly, genes encoding for TREX1 and RNASEH2 are also mutated in the Aicardi-Goutière syndrome (AGS), highlighting how these proteins provide a link between autoimmunity and nucleic acid metabolism [62,63,86-88]. This is also supported by their capacity to control endogenous retrotransposition [89-91]. An interesting possibility is that endogenous retroelements, alike abnormal replication intermediates, could trigger the activation of SLX4, a mechanism that would in turn lead to chronic inflammation in FA disease.

Once activated, how does SLX4 distinguish “good DNA” that will lead to productive infection, from “bad DNA” that will be destroyed? Such a question has already been brought up regarding TREX1 [92]. One hypothesis is that only non-productive short DNA generated by errors during reverse transcription may be targeted by nucleases. Alternatively, access to the viral DNA may be governed by the stability of the capsid core that is implicated in the control of reverse transcription [93-95]. In any event, identification of immune sensors associated to this process should improve our understanding of how HIV-1 escapes from the innate immune response. Whether the recently identified DNA sensors of HIV are specifically recruited in the absence of Vpr but not in its presence should be further addressed [78,96,97].

Another intriguing question concerns the presence or not of a signature of positive selection in SLX4. Such signature characterizes restriction factors and corresponds to variations in their sequences at the interface with the viral protein, reflecting an evolutionary arms-race between the virus and its host [98]. Recent work has shown how these sequence variations contribute to host susceptibility to viral infection. Here regarding the interplay between Vpr and SLX4, we are facing a new situation since SLX4 is not an antiviral factor, but is rather beneficial for the virus. One may speculate that co-evolution would push SLX4 to avoid Vpr in order to restore an immune response, while Vpr would evolve to conserve its association with SLX4.

### Vpr, SLX4com and a cellular system relevant for Vpr’s study

Future work should aim at elucidating whether Vpr provides an advantage to viral infection by activating SLX4. Thus, an important issue is to analyze whether the delta-Vpr HIV-1 virus is similarly infectious in cells depleted or not of SLX4 as the wt virus in SLX4−/− cells. The limiting factor in such study is the choice of a cellular system in which Vpr would clearly increase viral infection. So far, macrophages have represented an appropriate system, since in these cells, Vpr-deficient HIV-1 showed a slight replication defect [9-12]. In contrast to previous findings, one study has also reported that Vpr is able to enhance HIV-1 infection in peripheral blood mononuclear cells and dendritic cells [99]. More studies are needed to assess what is the best *in vitro* cellular model in which replication would depend on the presence of Vpr. In macrophages, the question of the presence of SLX4 and the associated nucleases has to be addressed before wondering whether Vpr-mediated enhancement of macrophage infection is linked to the activity of SLX4com. Of note, Zimmerman *et al.* previously found that HIV-1 Vpr fails to induce activation of the ATR pathway in macrophages, due to the lack of protein expression of at least ATR, Chk1 and Rad17 [19]. However, the question is still open regarding SLX4com. If SLX4 drives HIV-1 Vpr function in certain cell types such as macrophages, then, one would like to know whether differences of Vpr alleles to activate SLX4 correlate with different capabilities to help macrophage infection.

## Conclusions

The study from Laguette *et al.* provides important insights into the mechanism of Vpr-mediated G2 arrest with the identification of SLX4com as a new functional partner of Vpr. This discovery also opens new avenues to understand the biological role for Vpr along the viral life cycle. The idea that HIV may have developed a protein to induce the degradation of its own viral DNA leads to the intriguing questions of how the virus can achieve the right balance between preservation of viral DNA synthesis and escape from immune sensing through viral DNA degradation. In recent years, immune sensing has taken on a growing role in our understanding of the battle between the virus and the cell, making it important to identify the molecular players at stake such as SLX4com. Research associated to other diseases, namely AGS and FA, will certainly benefit from these discoveries.

## References

[1] Malim MH, Emerman M (2008). HIV-1 accessory proteins–ensuring viral survival in a hostile environment. Cell Host Microbe.

[2] Strebel K (2013). HIV accessory proteins versus host restriction factors. Curr Opin Virol.

[3] Ayinde D, Maudet C, Transy C, Margottin-Goguet F (2010). Limelight on two HIV/SIV accessory proteins in macrophage infection: is Vpx overshadowing Vpr?. Retrovirology.

[4] He J, Choe S, Walker R, Di Marzio P, Morgan DO, Landau NR (1995). Human immunodeficiency virus type 1 viral protein R (Vpr) arrests cells in the G2 phase of the cell cycle by inhibiting p34cdc2 activity. J Virol.

[5] Jowett JB, Planelles V, Poon B, Shah NP, Chen ML, Chen IS (1995). The human immunodeficiency virus type 1 vpr gene arrests infected T cells in the G2 + M phase of the cell cycle. J Virol.

[6] Planelles V, Bachelerie F, Jowett JB, Haislip A, Xie Y, Banooni P, Masuda T, Chen IS (1995). Fate of the human immunodeficiency virus type 1 provirus in infected cells: a role for vpr. J Virol.

[7] Re F, Braaten D, Franke EK, Luban J (1995). Human immunodeficiency virus type 1 Vpr arrests the cell cycle in G2 by inhibiting the activation of p34cdc2-cyclin B. J Virol.

[8] Rogel ME, Wu LI, Emerman M (1995). The human immunodeficiency virus type 1 vpr gene prevents cell proliferation during chronic infection. J Virol.

[9] Balliet JW, Kolson DL, Eiger G, Kim FM, McGann KA, Srinivasan A, Collman R (1994). Distinct effects in primary macrophages and lymphocytes of the human immunodeficiency virus type 1 accessory genes vpr, vpu, and nef: mutational analysis of a primary HIV-1 isolate. Virology.

[10] Connor RI, Chen BK, Choe S, Landau NR (1995). Vpr is required for efficient replication of human immunodeficiency virus type-1 in mononuclear phagocytes. Virology.

[11] Eckstein DA, Sherman MP, Penn ML, Chin PS, De Noronha CM, Greene WC, Goldsmith MA (2001). HIV-1 Vpr enhances viral burden by facilitating infection of tissue macrophages but not nondividing CD4+ T cells. J Exp Med.

[12] Jacquot G, Le Rouzic E, David A, Mazzolini J, Bouchet J, Bouaziz S, Niedergang F, Pancino G, Benichou S (2007). Localization of HIV-1 Vpr to the nuclear envelope: impact on Vpr functions and virus replication in macrophages. Retrovirology.

[13] Laguette N, Bregnard C, Hue P, Basbous J, Yatim A, Larroque M, Kirchhoff F, Constantinou A, Sobhian B, Benkirane M (2014). Premature Activation of the SLX4 Complex by Vpr Promotes G2/M Arrest and Escape from Innate Immune Sensing. Cell.

[14] Andersen JL, Planelles V (2005). The role of Vpr in HIV-1 pathogenesis. Curr HIV Res.

[15] Lai M, Zimmerman ES, Planelles V, Chen J (2005). Activation of the ATR pathway by human immunodeficiency virus type 1 Vpr involves its direct binding to chromatin in vivo. J Virol.

[16] Roshal M, Kim B, Zhu Y, Nghiem P, Planelles V (2003). Activation of the ATR-mediated DNA damage response by the HIV-1 viral protein R. J Biol Chem.

[17] Ward J, Davis Z, DeHart J, Zimmerman E, Bosque A, Brunetta E, Mavilio D, Planelles V, Barker E (2009). HIV-1 Vpr triggers natural killer cell-mediated lysis of infected cells through activation of the ATR-mediated DNA damage response. PLoS Pathog.

[18] Zimmerman ES, Chen J, Andersen JL, Ardon O, Dehart JL, Blackett J, Choudhary SK, Camerini D, Nghiem P, Planelles V (2004). Human immunodeficiency virus type 1 Vpr-mediated G2 arrest requires Rad17 and Hus1 and induces nuclear BRCA1 and gamma-H2AX focus formation. Mol Cell Biol.

[19] Zimmerman ES, Sherman MP, Blackett JL, Neidleman JA, Kreis C, Mundt P, Williams SA, Warmerdam M, Kahn J, Hecht FM, Grant RM, de Noronha CM, Weyrich AS, Greene WC, Planelles V (2006). Human immunodeficiency virus type 1 Vpr induces DNA replication stress in vitro and in vivo. J Virol.

[20] Zeman MK, Cimprich KA (2014). Causes and consequences of replication stress. Nat Cell Biol.

[21] Andersen JL, Zimmerman ES, DeHart JL, Murala S, Ardon O, Blackett J, Chen J, Planelles V (2005). ATR and GADD45alpha mediate HIV-1 Vpr-induced apoptosis. Cell Death Differ.

[22] Belzile JP, Duisit G, Rougeau N, Mercier J, Finzi A, Cohen EA (2007). HIV-1 Vpr-mediated G2 arrest involves the DDB1-CUL4AVPRBP E3 ubiquitin ligase. PLoS Pathog.

[23] Li G, Park HU, Liang D, Zhao RY (2010). Cell cycle G2/M arrest through an S phase-dependent mechanism by HIV-1 viral protein R. Retrovirology.

[24] DeHart JL, Zimmerman ES, Ardon O, Monteiro-Filho CM, Arganaraz ER, Planelles V (2007). HIV-1 Vpr activates the G2 checkpoint through manipulation of the ubiquitin proteasome system. Virol J.

[25] Hrecka K, Gierszewska M, Srivastava S, Kozaczkiewicz L, Swanson SK, Florens L, Washburn MP, Skowronski J (2007). Lentiviral Vpr usurps Cul4-DDB1[VprBP] E3 ubiquitin ligase to modulate cell cycle. Proc Natl Acad Sci U S A.

[26] Le Rouzic E, Belaidouni N, Estrabaud E, Morel M, Rain JC, Transy C, Margottin-Goguet F (2007). HIV1 Vpr arrests the cell cycle by recruiting DCAF1/VprBP, a receptor of the Cul4-DDB1 ubiquitin ligase. Cell Cycle.

[27] Schrofelbauer B, Hakata Y, Landau NR (2007). HIV-1 Vpr function is mediated by interaction with the damage-specific DNA-binding protein DDB1. Proc Natl Acad Sci U S A.

[28] Tan L, Ehrlich E, Yu XF (2007). DDB1 and Cul4A are required for human immunodeficiency virus type 1 Vpr-induced G2 arrest. J Virol.

[29] Wen X, Duus KM, Friedrich TD, de Noronha CM (2007). The HIV1 protein Vpr acts to promote G2 cell cycle arrest by engaging a DDB1 and Cullin4A-containing ubiquitin ligase complex using VprBP/DCAF1 as an adaptor. J Biol Chem.

[30] Zhao LJ, Mukherjee S, Narayan O (1994). Biochemical mechanism of HIV-I Vpr function. Specific interaction with a cellular protein. J Biol Chem.

[31] Angers S, Li T, Yi X, MacCoss MJ, Moon RT, Zheng N (2006). Molecular architecture and assembly of the DDB1-CUL4A ubiquitin ligase machinery. Nature.

[32] He YJ, McCall CM, Hu J, Zeng Y, Xiong Y (2006). DDB1 functions as a linker to recruit receptor WD40 proteins to CUL4-ROC1 ubiquitin ligases. Genes Dev.

[33] Higa LA, Zhang H (2007). Stealing the spotlight: CUL4-DDB1 ubiquitin ligase docks WD40-repeat proteins to destroy. Cell Div.

[34] Jin J, Arias EE, Chen J, Harper JW, Walter JC (2006). A family of diverse Cul4-Ddb1-interacting proteins includes Cdt2, which is required for S phase destruction of the replication factor Cdt1. Mol Cell.

[35] Le Rouzic E, Morel M, Ayinde D, Belaidouni N, Letienne J, Transy C, Margottin-Goguet F (2008). Assembly with the Cul4A-DDB1DCAF1 ubiquitin ligase protects HIV-1 Vpr from proteasomal degradation. J Biol Chem.

[36] Kim Y, Lach FP, Desetty R, Hanenberg H, Auerbach AD, Smogorzewska A (2011). Mutations of the SLX4 gene in Fanconi anemia. Nat Genet.

[37] Stoepker C, Hain K, Schuster B, Hilhorst-Hofstee Y, Rooimans MA, Steltenpool J, Oostra AB, Eirich K, Korthof ET, Nieuwint AW, Jaspers NG, Bettecken T, Joenje H, Schindler D, Rouse J, de Winter JP (2011). SLX4, a coordinator of structure-specific endonucleases, is mutated in a new Fanconi anemia subtype. Nat Genet.

[38] Munoz IM, Hain K, Declais AC, Gardiner M, Toh GW, Sanchez-Pulido L, Heuckmann JM, Toth R, Macartney T, Eppink B, Kanaar R, Ponting CP, Lilley DM, Rouse J (2009). Coordination of structure-specific nucleases by human SLX4/BTBD12 is required for DNA repair. Mol Cell.

[39] Svendsen JM, Smogorzewska A, Sowa ME, O’Connell BC, Gygi SP, Elledge SJ, Harper JW (2009). Mammalian BTBD12/SLX4 assembles a Holliday junction resolvase and is required for DNA repair. Cell.

[40] Fekairi S, Scaglione S, Chahwan C, Taylor ER, Tissier A, Coulon S, Dong MQ, Ruse C, Yates JR, Russell P, Fuchs RP, McGowan CH, Gaillard PH (2009). Human SLX4 is a Holliday junction resolvase subunit that binds multiple DNA repair/recombination endonucleases. Cell.

[41] Wyatt HD, Sarbajna S, Matos J, West SC (2013). Coordinated actions of SLX1-SLX4 and MUS81-EME1 for Holliday junction resolution in human cells. Mol Cell.

[42] Garner E, Kim Y, Lach FP, Kottemann MC, Smogorzewska A (2013). Human GEN1 and the SLX4-associated nucleases MUS81 and SLX1 are essential for the resolution of replication-induced Holliday junctions. Cell Rep.

[43] Castor D, Nair N, Declais AC, Lachaud C, Toth R, Macartney TJ, Lilley DM, Arthur JS, Rouse J (2013). Cooperative control of holliday junction resolution and DNA repair by the SLX1 and MUS81-EME1 nucleases. Mol Cell.

[44] Dehe PM, Coulon S, Scaglione S, Shanahan P, Takedachi A, Wohlschlegel JA, Yates JR, Llorente B, Russell P, Gaillard PH (2013). Regulation of Mus81-Eme1 Holliday junction resolvase in response to DNA damage. Nat Struct Mol Biol.

[45] Gallo-Fernandez M, Saugar I, Ortiz-Bazan MA, Vazquez MV, Tercero JA (2012). Cell cycle-dependent regulation of the nuclease activity of Mus81-Eme1/Mms4. Nucleic Acids Res.

[46] Matos J, Blanco MG, Maslen S, Skehel JM, West SC (2011). Regulatory control of the resolution of DNA recombination intermediates during meiosis and mitosis. Cell.

[47] Matos J, Blanco MG, West SC (2013). Cell-cycle kinases coordinate the resolution of recombination intermediates with chromosome segregation. Cell Rep.

[48] Saugar I, Vazquez MV, Gallo-Fernandez M, Ortiz-Bazan MA, Segurado M, Calzada A, Tercero JA (2013). Temporal regulation of the Mus81-Mms4 endonuclease ensures cell survival under conditions of DNA damage. Nucleic Acids Res.

[49] Berger G, Lawrence M, Hue S, Neil SJ: G2/M cell cycle arrest correlates with primate lentiviral Vpr interaction with the SLX4 complex. *J Virol* 2014. 10.1128/JVI.02307-14 PMC4301105 25320300

[50] Kim H, D’Andrea AD (2012). Regulation of DNA cross-link repair by the Fanconi anemia/BRCA pathway. Genes Dev.

[51] Tachiwana H, Shimura M, Nakai-Murakami C, Tokunaga K, Takizawa Y, Sata T, Kurumizaka H, Ishizaka Y (2006). HIV-1 Vpr induces DNA double-strand breaks. Cancer Res.

[52] Hanada K, Budzowska M, Davies SL, van Drunen E, Onizawa H, Beverloo HB, Maas A, Essers J, Hickson ID, Kanaar R (2007). The structure-specific endonuclease Mus81 contributes to replication restart by generating double-strand DNA breaks. Nat Struct Mol Biol.

[53] Liu B, Woltman AM, Janssen HL, Boonstra A (2009). Modulation of dendritic cell function by persistent viruses. J Leukoc Biol.

[54] Doehle BP, Hladik F, McNevin JP, McElrath MJ, Gale M (2009). Human immunodeficiency virus type 1 mediates global disruption of innate antiviral signaling and immune defenses within infected cells. J Virol.

[55] Okumura A, Alce T, Lubyova B, Ezelle H, Strebel K, Pitha PM (2008). HIV-1 accessory proteins VPR and Vif modulate antiviral response by targeting IRF-3 for degradation. Virology.

[56] Majumder B, Venkatachari NJ, Srinivasan A, Ayyavoo V (2009). HIV-1 mediated immune pathogenesis: spotlight on the role of viral protein R (Vpr). Curr HIV Res.

[57] Sato K, Misawa N, Iwami S, Satou Y, Matsuoka M, Ishizaka Y, Ito M, Aihara K, An DS, Koyanagi Y (2013). HIV-1 Vpr accelerates viral replication during acute infection by exploitation of proliferating CD4+ T cells in vivo. PLoS Pathog.

[58] Butler SL, Hansen MS, Bushman FD (2001). A quantitative assay for HIV DNA integration in vivo. Nat Med.

[59] Yan N, Regalado-Magdos AD, Stiggelbout B, Lee-Kirsch MA, Lieberman J (2010). The cytosolic exonuclease TREX1 inhibits the innate immune response to human immunodeficiency virus type 1. Nat Immunol.

[60] Zahoor MA, Xue G, Sato H, Murakami T, Takeshima SN, Aida Y (2014). HIV-1 Vpr Induces Interferon-Stimulated Genes in Human Monocyte-Derived Macrophages. PLoS One.

[61] Lim ES, Fregoso OI, McCoy CO, Matsen FA, Malik HS, Emerman M (2012). The ability of primate lentiviruses to degrade the monocyte restriction factor SAMHD1 preceded the birth of the viral accessory protein Vpx. Cell Host Microbe.

[62] Hrecka K, Hao C, Gierszewska M, Swanson SK, Kesik-Brodacka M, Srivastava S, Florens L, Washburn MP, Skowronski J (2011). Vpx relieves inhibition of HIV-1 infection of macrophages mediated by the SAMHD1 protein. Nature.

[63] Laguette N, Sobhian B, Casartelli N, Ringeard M, Chable-Bessia C, Segeral E, Yatim A, Emiliani S, Schwartz O, Benkirane M (2011). SAMHD1 is the dendritic- and myeloid-cell-specific HIV-1 restriction factor counteracted by Vpx. Nature.

[64] Clifford R, Louis T, Robbe P, Ackroyd S, Burns A, Timbs AT, Wright Colopy G, Dreau H, Sigaux F, Judde JG, Rotger M, Telenti A, Lin YL, Pasero P, Maelfait J, Titsias M, Cohen DR, Henderson SJ, Ross MT, Bentley D, Hillmen P, Pettitt A, Rehwinkel J, Knight SJ, Taylor JC, Crow YJ, Benkirane M, Schuh A (2014). SAMHD1 is mutated recurrently in chronic lymphocytic leukemia and is involved in response to DNA damage. Blood.

[65] Rice GI, Bond J, Asipu A, Brunette RL, Manfield IW, Carr IM, Fuller JC, Jackson RM, Lamb T, Briggs TA, Ali M, Gornall H, Couthard LR, Aeby A, Attard-Montalto SP, Bertini E, Bodemer C, Brockmann K, Brueton LA, Corry PC, Desguerre I, Fazzi E, Cazorla AG, Gener B, Hamel BC, Heiberg A, Hunter M, van der Knaap MS, Kumar R, Lagae L (2009). Mutations involved in Aicardi-Goutieres syndrome implicate SAMHD1 as regulator of the innate immune response. Nat Genet.

[66] Berger A, Sommer AF, Zwarg J, Hamdorf M, Welzel K, Esly N, Panitz S, Reuter A, Ramos I, Jatiani A, Mulder LC, Fernandez-Sesma A, Rutsch F, Simon V, König R, Flory E (2011). SAMHD1-deficient CD14+ cells from individuals with Aicardi-Goutieres syndrome are highly susceptible to HIV-1 infection. PLoS Pathog.

[67] Manel N, Hogstad B, Wang Y, Levy DE, Unutmaz D, Littman DR (2010). A cryptic sensor for HIV-1 activates antiviral innate immunity in dendritic cells. Nature.

[68] Pertel T, Reinhard C, Luban J (2011). Vpx rescues HIV-1 transduction of dendritic cells from the antiviral state established by type 1 interferon. Retrovirology.

[69] Puigdomenech I, Casartelli N, Porrot F, Schwartz O (2013). SAMHD1 restricts HIV-1 cell-to-cell transmission and limits immune detection in monocyte-derived dendritic cells. J Virol.

[70] Gao D, Wu J, Wu YT, Du F, Aroh C, Yan N, Sun L, Chen ZJ (2013). Cyclic GMP-AMP synthase is an innate immune sensor of HIV and other retroviruses. Science.

[71] Baldauf HM, Pan X, Erikson E, Schmidt S, Daddacha W, Burggraf M, Schenkova K, Ambiel I, Wabnitz G, Gramberg T, Panitz S, Flory E, Landau NR, Sertel S, Rutsch F, Lasitschka F, Kim B, König R, Fackler OT, Keppler OT (2012). SAMHD1 restricts HIV-1 infection in resting CD4(+) T cells. Nat Med.

[72] Beloglazova N, Flick R, Tchigvintsev A, Brown G, Popovic A, Nocek B, Yakunin AF (2013). Nuclease Activity of the Human SAMHD1 Protein Implicated in the Aicardi-Goutieres Syndrome and HIV-1 Restriction. J Biol Chem.

[73] Descours B, Cribier A, Chable-Bessia C, Ayinde D, Rice G, Crow Y, Yatim A, Schwartz O, Laguette N, Benkirane M (2012). SAMHD1 restricts HIV-1 reverse transcription in quiescent CD4+ T-cells. Retrovirology.

[74] Goldstone DC, Ennis-Adeniran V, Hedden JJ, Groom HC, Rice GI, Christodoulou E, Walker PA, Kelly G, Haire LF, Yap MW, de Carvalho LP, Stoye JP, Crow YJ, Taylor IA, Webb M (2011). HIV-1 restriction factor SAMHD1 is a deoxynucleoside triphosphate triphosphohydrolase. Nature.

[75] Lahouassa H, Daddacha W, Hofmann H, Ayinde D, Logue EC, Dragin L, Bloch N, Maudet C, Bertrand M, Gramberg T, Pancino G, Priet S, Canard B, Laguette N, Benkirane M, Transy C, Landau NR, Kim B, Margottin-Goguet F (2012). SAMHD1 restricts the replication of human immunodeficiency virus type 1 by depleting the intracellular pool of deoxynucleoside triphosphates. Nat Immunol.

[76] Powell RD, Holland PJ, Hollis T, Perrino FW (2011). Aicardi-Goutieres syndrome gene and HIV-1 restriction factor SAMHD1 is a dGTP-regulated deoxynucleotide triphosphohydrolase. J Biol Chem.

[77] Ryoo J, Choi J, Oh C, Kim S, Seo M, Kim SY, Seo D, Kim J, White TE, Brandariz-Nunez A, Nuñez A, Diaz-Griffero F, Yun CH, Hollenbaugh JA, Kim B, Baek D, Ahn K (2014). The ribonuclease activity of SAMHD1 is required for HIV-1 restriction. Nat Med.

[78] Lahaye X, Satoh T, Gentili M, Cerboni S, Conrad C, Hurbain I, El Marjou A, Lacabaratz C, Lelievre JD, Manel N (2013). The capsids of HIV-1 and HIV-2 determine immune detection of the viral cDNA by the innate sensor cGAS in dendritic cells. Immunity.

[79] Rasaiyaah J, Tan CP, Fletcher AJ, Price AJ, Blondeau C, Hilditch L, Jacques DA, Selwood DL, James LC, Noursadeghi M, Towers GJ (2013). HIV-1 evades innate immune recognition through specific cofactor recruitment. Nature.

[80] Landau NR (2014). The innate immune response to HIV-1: to sense or not to sense. DNA Cell Biol.

[81] Rowland-Jones SL, Whittle HC (2007). Out of Africa: what can we learn from HIV-2 about protective immunity to HIV-1?. Nat Immunol.

[82] Bregnard C, Benkirane M, Laguette N (2014). DNA damage repair machinery and HIV escape from innate immune sensing. Front Microbiol.

[83] Gehrke N, Mertens C, Zillinger T, Wenzel J, Bald T, Zahn S, Tuting T, Hartmann G, Barchet W (2013). Oxidative damage of DNA confers resistance to cytosolic nuclease TREX1 degradation and potentiates STING-dependent immune sensing. Immunity.

[84] Haruki M, Tsunaka Y, Morikawa M, Kanaya S (2002). Cleavage of a DNA-RNA-DNA/DNA chimeric substrate containing a single ribonucleotide at the DNA-RNA junction with prokaryotic RNases HII. FEBS Lett.

[85] Yang YG, Lindahl T, Barnes DE (2007). Trex1 exonuclease degrades ssDNA to prevent chronic checkpoint activation and autoimmune disease. Cell.

[86] Crow YJ, Hayward BE, Parmar R, Robins P, Leitch A, Ali M, Black DN, van Bokhoven H, Brunner HG, Hamel BC, Corry PC, Cowan FM, Frints SG, Klepper J, Livingston JH, Lynch SA, Massey RF, Meritet JF, Michaud JL, Ponsot G, Voit T, Lebon P, Bonthron DT, Jackson AP, Barnes DE, Lindahl T (2006). Mutations in the gene encoding the 3′-5′ DNA exonuclease TREX1 cause Aicardi-Goutieres syndrome at the AGS1 locus. Nat Genet.

[87] Rice G, Newman WG, Dean J, Patrick T, Parmar R, Flintoff K, Robins P, Harvey S, Hollis T, O’Hara A, Herrick AL, Bowden AP, Perrino FW, Lindahl T, Barnes DE, Crow YJ (2007). Heterozygous mutations in TREX1 cause familial chilblain lupus and dominant Aicardi-Goutieres syndrome. Am J Hum Genet.

[88] Rice G, Patrick T, Parmar R, Taylor CF, Aeby A, Aicardi J, Artuch R, Montalto SA, Bacino CA, Barroso B, Baxter P, Benko WS, Bergmann C, Bertini E, Biancheri R, Blair EM, Blau N, Bonthron DT, Briggs T, Brueton LA, Brunner HG, Burke CJ, Carr IM, Carvalho DR, Chandler KE, Christen HJ, Corry PC, Cowan FM, Cox H, D’Arrigo S (2007). Clinical and molecular phenotype of Aicardi-Goutieres syndrome. Am J Hum Genet.

[89] Stetson DB, Ko JS, Heidmann T, Medzhitov R (2008). Trex1 prevents cell-intrinsic initiation of autoimmunity. Cell.

[90] Esnault C, Millet J, Schwartz O, Heidmann T (2006). Dual inhibitory effects of APOBEC family proteins on retrotransposition of mammalian endogenous retroviruses. Nucleic Acids Res.

[91] Zhao K, Du J, Han X, Goodier JL, Li P, Zhou X, Wei W, Evans SL, Li L, Zhang W, Cheung LE, Wang G, Kazazian HH, Yu XF (2013). Modulation of LINE-1 and Alu/SVA retrotransposition by Aicardi-Goutieres syndrome-related SAMHD1. Cell Rep.

[92] Hasan M, Yan N (2014). Safeguard against DNA sensing: the role of TREX1 in HIV-1 infection and autoimmune diseases. Front Microbiol.

[93] Ambrose Z, Lee K, Ndjomou J, Xu H, Oztop I, Matous J, Takemura T, Unutmaz D, Engelman A, Hughes SH, KewalRamani VN (2012). Human immunodeficiency virus type 1 capsid mutation N74D alters cyclophilin A dependence and impairs macrophage infection. J Virol.

[94] Tang S, Murakami T, Agresta BE, Campbell S, Freed EO, Levin JG (2001). Human immunodeficiency virus type 1 N-terminal capsid mutants that exhibit aberrant core morphology and are blocked in initiation of reverse transcription in infected cells. J Virol.

[95] Yang Y, Fricke T, Diaz-Griffero F (2013). Inhibition of reverse transcriptase activity increases stability of the HIV-1 core. J Virol.

[96] Monroe KM, Yang Z, Johnson JR, Geng X, Doitsh G, Krogan NJ, Greene WC (2014). IFI16 DNA sensor is required for death of lymphoid CD4 T cells abortively infected with HIV. Science.

[97] Jakobsen MR, Bak RO, Andersen A, Berg RK, Jensen SB, Tengchuan J, Laustsen A, Hansen K, Ostergaard L, Fitzgerald KA, Xiao TS, Mikkelsen JG, Mogensen TH, Paludan SR (2013). IFI16 senses DNA forms of the lentiviral replication cycle and controls HIV-1 replication. Proc Natl Acad Sci U S A.

[98] Duggal NK, Emerman M (2012). Evolutionary conflicts between viruses and restriction factors shape immunity. Nat Rev Immunol.

[99] de Silva S, Planelles V, Wu L (2012). Differential effects of Vpr on single-cycle and spreading HIV-1 infections in CD4+ T-cells and dendritic cells. PLoS One.

